# Therapeutic Exercise for Chronic Whiplash-Associated Disorders: A Systematic Review of Randomized Controlled Trials on Pain, Disability, and Kinesiophobia

**DOI:** 10.3390/diseases14100366

**Published:** 2026-10-02

**Authors:** Rosario Ferlito, Giuseppe Lanza, Gianluca Testa, Vito Pavone, Fabio Manfrè, Salvatore Giunta

**Affiliations:** 1 Department of Biomedical and Biotechnological Sciences, University of Catania, Via Santa Sofia 97, 95123 Catania, Italy; ferlito.rosario@libero.it (R.F.); fabiomanfre94@gmail.com (F.M.); sgiunta@unict.it (S.G.); 2 Department of Surgery and Medical-Surgical Specialties, University of Catania, Via Santa Sofia 78, 95123 Catania, Italy; gianluca.testa@unict.it (G.T.); vito.pavone@unict.it (V.P.); 3 Clinical Neurophysiology Research Unit, Oasi Research Institute-IRCCS, Via Conte Ruggero, 73, 94018 Troina, Italy; 4 A.O.U. Policlinico Rodolico-San Marco, University of Catania, Via Santa Sofia 78, 95123 Catania, Italy

**Keywords:** whiplash-associated disorders, therapeutic exercise, neck pain, disability, kinesiophobia, rehabilitation

## Abstract

Background/Objectives: Chronic whiplash-associated disorders (WAD) are a common cause of persistent neck pain and disability. Therapeutic exercise is widely used, but its effectiveness remains uncertain. This systematic review evaluated the effects of therapeutic exercise, alone or combined with other interventions, on pain, neck-related disability, and kinesiophobia in adults with chronic WAD. Methods: PubMed, PEDro, and the Cochrane Library were searched from inception to December 2025 for randomized controlled trials (RCTs) including adults with chronic WAD (>3 months). Eligible studies compared exercise-based interventions with minimal intervention, usual care, or other active treatments. Primary outcomes were pain and neck-related disability, with kinesiophobia as a secondary outcome. Methodological quality was assessed using the PEDro scale, risk of bias using Cochrane RoB 2, and certainty of evidence using GRADE. Due to heterogeneity, findings were synthesized without meta-analysis using SWiM principles. Results: Six reports representing five independent RCT cohorts (up to 635 unique participants) were included. Interventions included neck-specific exercise, home-based programmes, multimodal rehabilitation, and exercise combined with manual therapy. Exercise generally produced greater short- to medium-term improvements in pain and disability than minimal intervention or advice, whereas comparisons with other active treatments were heterogeneous and statistically inconclusive. Effects on kinesiophobia were inconsistent. PEDro scores ranged from 7–9/10. RoB 2 indicated predominantly some concerns. GRADE certainty was low for pain and disability and very low for kinesiophobia. Conclusions: Therapeutic exercise may improve pain and disability compared with minimal interventions, but evidence certainty is low. Its superiority over other active treatments and effects on kinesiophobia remain uncertain.

## 1. Introduction

Whiplash-associated disorders (WAD) are a frequent consequence of acceleration–deceleration injuries of the cervical spine, most commonly following motor vehicle collisions [1,2]. Epidemiologically, WAD represents a relevant global health burden, although its frequency varies considerably across geographical regions and healthcare systems. Its annual incidence has been estimated at approximately 300 cases per 100,000 inhabitants in Western populations, and a substantial proportion of affected individuals may progress to persistent or chronic symptoms [3,4]. While many individuals recover within the first three months after injury, a substantial proportion develop chronic symptoms, including persistent neck pain, stiffness, headaches, dizziness, and reduced functional capacity [1,4,5]. Chronic WAD thus imposes a considerable personal and socioeconomic burden, with high healthcare utilization, reduced work participation, and impaired quality of life [4,6].

Exercise-based rehabilitation is commonly applied in the management of neck pain and WAD in clinical practice. Therapeutic exercise encompasses different modalities, including isometric exercises, involving muscle contraction without joint movement; isotonic exercises, involving dynamic muscle contraction against resistance; and motor control exercises, aimed at improving coordination, movement quality, and neuromuscular control [7]. Therapeutic exercise may encompass neck-specific strengthening and endurance training, range-of-motion and flexibility exercises, postural and motor control training, and global physical activity. However, the optimal content, intensity, and duration of exercise programmes for chronic WAD remain debated, and clinical outcomes across trials are heterogeneous [8,9,10].

Beyond pain and physical impairment, several psychosocial factors, such as fear of movement, catastrophizing, and maladaptive coping strategies, among others, contribute to symptom persistence and disability after whiplash injury [11,12,13,14,15,16]. Kinesiophobia, defined as an excessive and irrational fear of physical movement and activity, has been identified as a potential mediator between pain and disability in chronic musculoskeletal conditions, including WAD [9,10,12,16]. Exercise programmes that explicitly address fear of movement and promote self-efficacy may therefore provide additional benefits compared with purely biomechanical approaches [17,18].

In this context, several randomized controlled trials (RCTs) have examined the effects of therapeutic exercise in individuals with chronic WAD, although findings vary in terms of magnitude and durability of treatment effects [8,9,10]. Moreover, to date, kinesiophobia and other psychological constructs are not consistently included as outcome measures, limiting the understanding of how exercise interventions may influence the broader biopsychosocial profile of these patients [9,10].

Previous systematic reviews have evaluated exercise therapy for WAD, reporting heterogeneous and generally low-certainty effects on pain and disability [19,20]. More recent evidence syntheses have examined exercise combined with educational or behavioral approaches and similarly emphasize heterogeneity and uncertainty regarding clinically important between-group effects [21]. However, the effects of therapeutic exercise, specifically in chronic WAD, while simultaneously considering pain, disability, and kinesiophobia, remain insufficiently characterized. The present review addresses this gap by focusing on RCTs of adults with chronic WAD and integrating these three clinically relevant outcomes. Therefore, a synthesis of available RCTs is needed to clarify the effectiveness of therapeutic exercise on pain, disability, and kinesiophobia in chronic WAD patients and to guide both clinical decision-making and the design of future studies.

Accordingly, using the PICO framework, this systematic review aimed to determine whether, in adults with chronic WAD (Population), therapeutic exercise, alone or combined with other interventions (Intervention), compared with minimal intervention, usual care, or other active treatments (Comparison), improves pain and neck-related disability and reduces kinesiophobia (Outcomes).

## 2. Materials and Methods

### 2.1. Study Design and Reporting

This systematic review was conducted and reported in accordance with the Preferred Reporting Items for Systematic Reviews and Meta-Analyses (PRISMA) 2020 statement [22]. The completed PRISMA 2020 checklist is provided as Supplementary Material (Table S1). The review question was framed using a Population–Intervention–Comparison–Outcome (PICO) approach [23]. The review protocol was not prospectively registered (e.g., in PROSPERO); the implications of the absence of prospective registration, including the potential risk of selective eligibility and outcome reporting, are acknowledged in the Limitations section.

### 2.2. Eligibility Criteria

- Population: RCTs were eligible if they included adults (≥18 years), predominantly young to middle-aged, with chronic WAD, defined as neck pain and related symptoms persisting for more than three months after a whiplash injury [1,4].
- Intervention: therapeutic exercise delivered as a stand-alone programme or as a core component of a multimodal rehabilitation approach, with a minimum intervention duration of 4 weeks [8,9,10,17].
- Comparison: minimal intervention, usual care, advice, or another active treatment.
- Outcomes: eligible trials were required to report at least one validated measure of pain intensity and/or neck-related disability. Kinesiophobia was considered a secondary outcome and was extracted and synthesized when reported, but its assessment was not required for study eligibility. Trials were excluded if they did not report any relevant clinical outcome for pain and/or neck-related disability.

### 2.3. Search Strategy

A literature search was performed in PubMed, Physiotherapy Evidence Database (PEDro), and the Cochrane Library from database inception to December 2025. Following revision, the search strategy was broadened to combine terms related to whiplash-associated disorders, chronic symptoms, therapeutic exercise, and randomized controlled trials, without restricting retrieval according to specific outcomes. In particular, kinesiophobia was not included as a mandatory search concept because it represented a secondary outcome and its inclusion could have reduced search sensitivity. Kinesiophobia-related data were instead extracted from eligible trials when reported. Reference lists of eligible articles and relevant reviews were also screened to identify additional studies. No language or publication date restrictions were applied. Table 1 reports the search strings used.

### 2.4. Study Selection and Extraction

**Figure 1:**
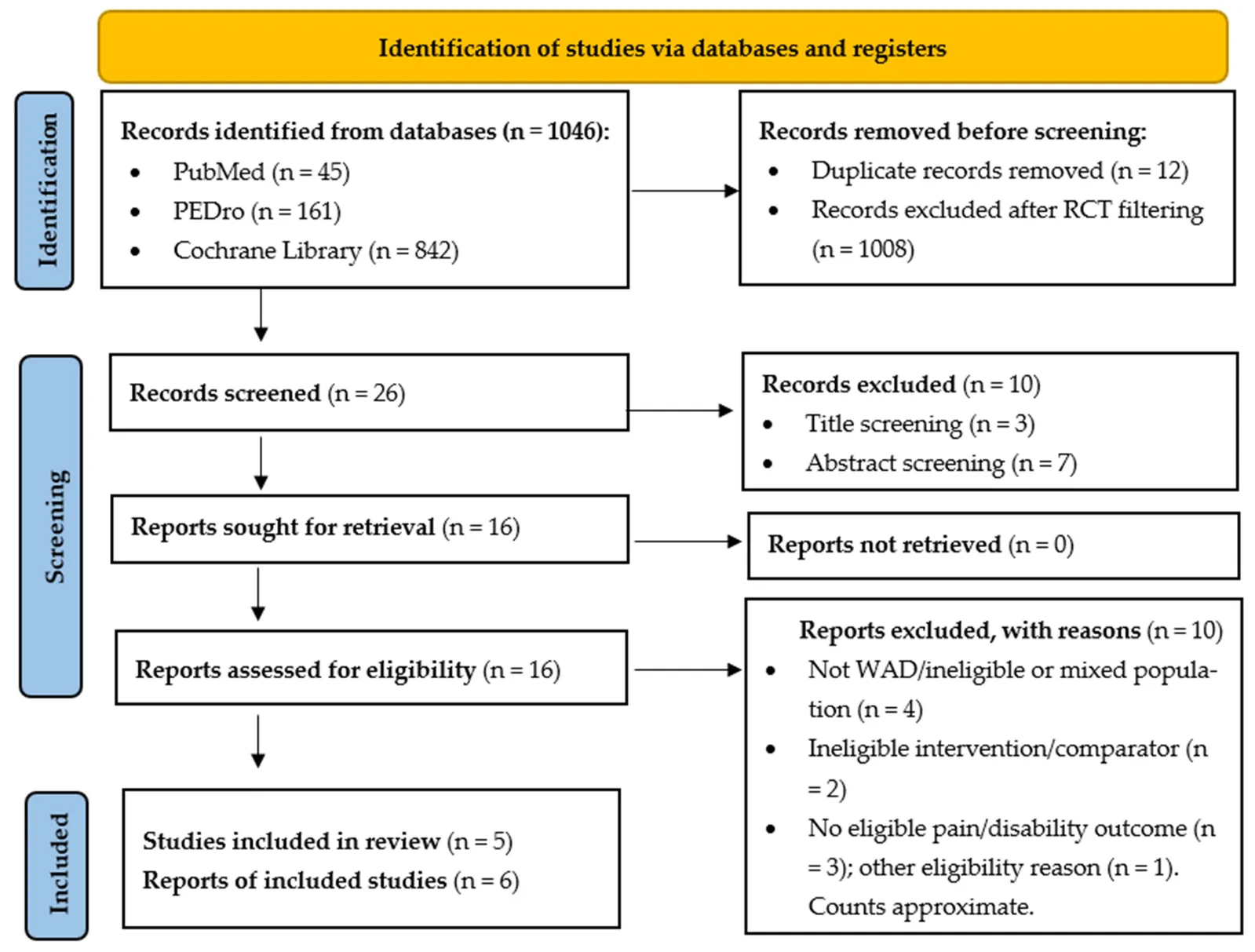
PRISMA flow diagram summarizing the study selection process (from [22]).

Titles and abstracts were independently screened by two reviewers (R.F. and G.L.). Disagreements were resolved through discussion and re-evaluation of the eligibility criteria. If consensus could not be reached, a third reviewer (S.G.) independently assessed the record and made the final decision. The same procedure was applied to full-text eligibility assessment. The study selection process is summarized in the PRISMA flow diagram (Figure 1). Data extraction was independently performed by two reviewers (R.F. and G.L.) using a standardized extraction form. Disagreements were resolved by the consensus of a third reviewer (S.G.), when required.

### 2.5. Methodological Quality (PEDro)

Methodological quality of the included trials was assessed using the PEDro scale [24], a validated tool that scores RCTs on a 0–10 scale based on methodological criteria including random allocation, concealed allocation, blinding, intention-to-treat analysis, adequacy of follow-up, and statistical reporting. Consistent with established thresholds, trials scoring 6–10 points were considered to have a good methodological quality, those scoring 4–5 a fair quality, and those scoring ≤ 3 a poor quality. Table 2 and Table 3 show the study quality according to PEDro scores and the quality categories of the included studies, respectively, whereas study characteristics are presented in Table 4.

In addition to the PEDro scale, risk of bias was assessed using the Cochrane Risk of Bias 2 (RoB 2) tool for randomized trials [25,26]. The five RoB 2 domains were considered: bias arising from the randomization process; bias due to deviations from intended interventions; bias due to missing outcome data; bias in measurement of the outcome; and bias in selection of the reported result. Judgements were classified as low risk of bias, some concerns, or high risk of bias. Because RoB 2 is outcome-specific, the assessment focused on the outcomes central to this review (pain/disability and, where reported, kinesiophobia). Two reviewers independently performed the assessments, with disagreements resolved by consensus with a third reviewer.

RoB 2 assessments were performed at the level of the relevant outcome/result rather than as a generic study-level quality rating. For each domain, judgments were based on the corresponding RoB 2 signalling questions and supported by trial-specific information concerning randomization and allocation concealment, deviations from intended interventions, completeness of outcome data, outcome measurement, and selection of the reported result. The evidence supporting each judgment is summarized in Table 5.

**Table 2:** Methodological quality of included studies assessed using the PEDro scale. ✓ = criterion satisfied; ✗ = criterion not satisfied.

Study [References]	1	2	3	4	5	6	7	8	9	10	11	PEDro Score
Sarig Bahat et al., 2018 * [8]	✓	✓	✓	✓	✗	✗	✗	✓	✓	✓	✓	7/10
Overmeer et al., 2016 [9]	✓	✓	✓	✓	✗	✗	✓	✓	✓	✓	✓	8/10
Peterson et al., 2015 [10]	✓	✓	✓	✓	✗	✗	✓	✓	✓	✓	✓	8/10
Javdaneh et al., 2020 [17]	✓	✓	✓	✓	✗	✗	✓	✓	✓	✓	✓	8/10
Thompson et al., 2016 * [27]	✓	✓	✓	✓	✗	✗	✗	✓	✓	✓	✓	7/10
Ris et al., 2016 * [28]	✓	✓	✓	✓	✗	✓	✓	✓	✓	✓	✓	9/10

Legend: PEDro = Physiotherapy Evidence Based Database. * Studies for which the PEDro score was not available in the PEDro database and was therefore independently assessed by the authors based on the information reported in the full text.

**Table 3:** Quality categories of the included studies (based on PEDro scores).

Quality Category (PEDro)	Studies	PEDro Score
Higher quality	Overmeer et al., 2016 [9] Peterson et al., 2015 [10] Javdaneh et al., 2020 [17] Ris et al., 2016 * [28]	8–9/10
Moderate quality	Sarig Bahat et al., 2018 * [8] Thompson et al., 2016 * [27]	7/10

Legend: PEDro = Physiotherapy Evidence Based Database. * Studies for which the PEDro score was not available in the PEDro database and was therefore independently assessed by the authors based on the information reported in the full text.

**Table 4:** Summary of the included studies.

Study [Refs]	Design	Participants	Interventions (Number of Treatments)	Outcomes	Follow-Up	Main Results	PEDro
Sarig Bahat et al., 2018 * [8]	RCT	n = 90; Age ≥ 18 years; Symptom duration > 3 months	Group 1: Virtual reality-based exercises (n = 30); Group 2: Laser-guided exercises (n = 30); Group 3: Control (n = 30); 4 weeks	NDI, GPE, cervical movement speed, VAS, TSK, EQ-5D	Baseline, 1 and 3 months	Both intervention groups showed significant improvements in NDI and cervical movement velocity compared with controls. VAS, GPE and EQ-5D improved at 3 months. No significant improvement in TSK was observed in Group 2.	7/10
Overmeer et al., 2016 [9]	RCT	n = 194; Age 18–63 years; Symptom duration ≥ 6 months and <3 years	Group 1: Neck-specific exercises (NSE; n = 67); Group 2: NSE + behavioral approach (NSEB; n = 68); Group 3: Prescribed physical activity (PPA; n = 59)	PDI, TSK, HAD, PCS (Pain Catastrophizing Scale)	Baseline, 3, 6, 12 and 24 months	General disability and pain decreased significantly in the NSEB group at 3 months and were maintained over time. NSE improved kinesiophobia and anxiety. Pain catastrophizing decreased in NSE and NSEB groups compared with PPA.	8/10
Peterson et al., 2015 [10]	RCT	n = 216; Age 18–63 years; Symptom duration ≥ 6 months and <3 years	Group 1: NSE (n = 76); Group 2: NSEB (n = 71); Group 3: PPA (n = 69)	Neck muscle endurance, TSK-11, VAS	Baseline, 3 and 6 months	Dorsal neck muscle endurance improved in NSE and NSEB compared with PPA. Pain intensity improved in NSE and NSEB. Only the NSE group showed significant improvements in kinesiophobia.	8/10
Javdaneh et al., 2020 [17]	RCT	n = 72; Age 20–45 years; Symptom duration ≥ 3 months	Group 1: Scapular exercises (n = 24); Group 2: Scapular exercises + cognitive functional therapy (n = 24); Group 3: Control (n = 24); 3 sessions/week for 6 weeks	VAS, TSK, muscle activity	Baseline, 6 weeks	Significant improvements in pain, kinesiophobia and muscle activity were observed in the experimental groups compared with controls, with greater effects in the multidisciplinary intervention.	8/10
Thompson et al., 2016 * [27]	RCT	n = 57; Symptom duration ≥ 3 months	Group 1: Progressive neck exercise program; Group 2: Interactive behavior modification therapy + neck exercises; 4 weekly sessions	NPQ, NPRS, TSK	Baseline, 6 months	No significant between-group difference was reported for disability. Pain reduction favored the behavioral intervention plus exercise group (*p* < 0.05); both groups improved over time.	7/10
Ris et al., 2016 * [28]	RCT	n = 200; Age ≥ 18 years; Symptom duration ≥ 6 months	Control: Pain education (n = 101); Intervention: Pain education + neck-specific exercises (n = 99); Education: 4 monthly sessions; Exercise: additional 8 sessions	SF-36 PCS, NDI, BDI-II, PSFS, TSK	Baseline, 4 months	Significant improvements were observed in all outcomes. The combined education and exercise group achieved greater benefits than education alone.	9/10

Legend: RCT = randomized controlled trial; TSK = Tampa Scale of Kinesiophobia; NDI = Neck Disability Index; VAS = Visual Analogue Scale; NPRS = numeric pain rating scale; NPQ = Northwick Park Neck Pain Questionnaire; GPE = global perceived effect; EQ-5D = EuroQol-5 Dimension; PDI = Pain Disability Index; HAD = Hospital Anxiety and Depression scale; PCS = Pain Catastrophizing Scale; SF-36 PCS = Short Form-36 Physical Component Summary; BDI-II = Beck Depression Inventory-II; PSFS = Patient-Specific Functional Scale. * PEDro score not available in the PEDro database and independently assessed by the authors based on the information reported in the full text.

**Table 5:** Risk of bias assessment of the independent randomized trial cohorts using the Cochrane RoB 2 tool.

Study	Randomization	Deviations	Missing Data	Outcome Measurement	Selective Reporting	Overall	Rationale for Judgment
Sarig Bahat et al., 2018 [8]	Some concerns	Low	Low	Some concerns	Some concerns	Some concerns	Randomized three-arm trial; participants and treating therapists could not be blinded because of the nature of the interventions. Attrition was relevant (90 randomized, 76 assessed at 1 month and 56 at 3 months), raising concerns regarding missing outcome data at follow-up. Outcomes were patient-reported and therefore potentially influenced by knowledge of treatment allocation.
Overmeer/Peterson trial, 2015–2016 [9,10]	Low	Low	Low	Some concerns	Some concerns	Some concerns	Random allocation and allocation concealment were reported, with blinded outcome assessors and intention-to-treat analysis. Participants and therapists were not blinded because of the exercise interventions. Patient-reported outcomes, including kinesiophobia and pain, remained susceptible to awareness of treatment allocation. This report derived from the same randomized multicentre cohort as Peterson et al. Allocation procedures and assessor blinding were reported, whereas participants and treating physiotherapists could not be blinded. Outcomes were self-administered, and some loss to follow-up occurred over the 24-month observation period; these features generated concerns particularly for outcome measurement and missing data.
Javdaneh et al., 2020 [17]	Some concerns	Low	Low	Some concerns	Some concerns	Some concerns	Single-blind randomized trial. Because the interventions were exercise/behavioral treatments, participants and therapists could not be blinded. Pain and kinesiophobia were self-reported outcomes and could therefore have been influenced by knowledge of intervention allocation; no clear evidence of major selective reporting was identified from the published report.
Thompson et al., 2016 [27]	Some concerns	Low	Some concerns	Some concerns	Some concerns	Some concerns	Randomized comparison of two active physiotherapy interventions; participant and therapist blinding was not feasible. Follow-up data were available for 39 of 57 randomized participants, generating concern regarding missing outcome data. Pain and disability were self-reported and potentially susceptible to knowledge of treatment allocation.
Ris et al., 2016 [28]	Low	Low	Low	Some concerns	Some concerns	Some concerns	Multicentre randomized trial with a prospectively registered protocol (NCT01431261). Participants and physiotherapists were aware of treatment allocation, whereas outcome assessors and data analysts were blinded. Several outcomes were patient-reported; comparison with the registered protocol reduced, but did not eliminate, concern regarding selective reporting.

Legend: RoB 2 = Cochrane Risk of Bias 2. Overmeer et al. (2016) [9] and Peterson et al. (2015) [10] were assessed as a single underlying trial cohort. Judgements refer to the principal patient-reported outcomes considered in this review.

### 2.6. Outcomes

The primary outcome was any change in pain intensity and neck-related disability from baseline to post-intervention and, when reported, to follow-up assessment(s). Pain was most commonly measured using a visual analogue scale (VAS) or numerical rating scale (NRS), while disability was typically assessed with the Neck Disability Index (NDI) or similar instruments. Kinesiophobia, when evaluated, was measured using validated questionnaires, such as the Tampa Scale for Kinesiophobia (TSK) [9,10,17,27,28]. Other secondary outcomes (e.g., quality of life, global perceived effect, return to work) were extracted but were beyond the focus of this review.

### 2.7. Data Summary

Given the clinical and methodological heterogeneity in exercise protocols, comparator interventions, outcome measures, and follow-up durations, quantitative pooling was not considered appropriate. In accordance with Synthesis Without Meta-analysis (SWiM) principles [29], studies were grouped by outcome domain (pain and disability), comparator type, and follow-up time point. Quantitative between-group effect estimates with 95% confidence intervals were extracted from the original reports whenever available; when these were not reported or could not be validly derived from the published data, this was indicated as not reported (NR). The synthesis considered the magnitude, direction, and precision of the observed effects rather than relying on statistical significance alone. Kinesiophobia, as a secondary outcome, was synthesized separately when reported. Quantitative between-group findings for the primary pain and disability outcomes are summarized in Supplementary Table S2.

### 2.8. Certainty of Evidence

The certainty of evidence for the primary outcomes (pain and neck-related disability) and the secondary outcome of kinesiophobia was assessed using the Grading of Recommendations Assessment, Development and Evaluation (GRADE) approach [30,31,32]. Certainty was rated as high, moderate, low, or very low considering risk of bias, inconsistency, indirectness, imprecision, and publication bias. Given the small number of independent trials and the heterogeneity of interventions, comparators, and outcome measures, downgrading decisions were made conservatively and are reflected in the narrative synthesis.

## 3. Results

### 3.1. Study Selection

The database search identified a total of 1046 records (PubMed n = 45; PEDro n = 161; Cochrane Library n = 842). After RCT filtering, 38 records remained. After removal of duplicates (n = 12), 26 records were screened by title and abstract; 10 were excluded (title screening n = 3; abstract screening n = 7), and 16 reports underwent full-text assessment. Ten full-text reports were excluded. Because the retained screening documentation did not preserve mutually exclusive reason codes for each excluded full-text report, the reason-specific counts were reconstructed retrospectively from the available screening notes and should be regarded as approximate: not WAD or an ineligible/mixed population without extractable WAD data (n = 4), ineligible intervention or comparator (n = 2), no eligible pain/disability outcome (n = 3), and other eligibility reason (n = 1). Six reports were included in the qualitative synthesis, representing five independent RCT cohorts. Overmeer et al. (2016) [9] and Peterson et al. (2015) [10] were reports from the same underlying randomized trial and were therefore treated as a single study cohort for participant counting. Multiple reports originating from the same underlying randomized cohort were treated as separate reports but not as independent studies, thereby avoiding double counting of participants and trial-level evidence.

### 3.2. Study Characteristics

The six included reports represented five independent RCT cohorts and up to 635 unique participants with chronic WAD. Overmeer et al. (2016) [9] and Peterson et al. (2015) [10] originated from the same underlying randomized trial; therefore, their samples were not summed when estimating the total number of unique participants. Individual study cohorts ranged from 57 to 216 participants. Most studies recruited adults with persistent neck pain and related symptoms following motor vehicle accidents, with symptom duration generally exceeding three months and often longer than six months. Exercise interventions varied considerably and included: supervised neck-specific exercise programmes; group-based or home-based exercise; systematic multimodal rehabilitation combining exercise with education, manual therapy or cognitive-behavioral elements; and combined exercise plus manual therapy delivered in physiotherapy settings. Comparators ranged from minimal intervention or usual care (e.g., advice only, information leaflets) to alternative active treatments such as self-management programmes or other physiotherapy approaches. Follow-up durations varied from immediate post-intervention to 12 months. Detailed study characteristics are summarized in Table 4.

### 3.3. Methodological Quality

PEDro scores of the included RCTs ranged from 7 to 9 out of 10 (Table 2), indicating overall good methodological quality. The complementary RoB 2 assessment nevertheless identified methodological concerns not fully captured by the PEDro score. Across the five independent RCT cohorts, the randomization process was generally judged at low risk or as raising some concerns when allocation concealment was insufficiently described. Because most clinically relevant outcomes were participant-reported and blinding of participants was generally not feasible, the measurement-of-outcome domain commonly raised some concerns. Missing outcome data were generally limited, although incomplete reporting in some trials led to some concerns. No trial was judged uniformly free of bias across all outcome domains. Overall, RoB 2 judgements were predominantly ‘some concerns’; these judgements were incorporated into the GRADE assessment.

Overall, the RoB 2 assessment identified predominantly some concerns rather than uniformly low risk of bias. The principal concerns were related to the unavoidable lack of participant and therapist blinding in exercise-based interventions, the use of self-reported pain, disability, and kinesiophobia outcomes, and incomplete outcome data in some trials, particularly at longer follow-up. For the selection-of-the-reported-result domain, judgments also considered whether trial registration, protocols, or sufficiently detailed prespecified analyses were available. When prespecification of the specific outcome result or analysis could not be verified, this uncertainty was reflected in the domain judgment rather than assuming a low risk of selective reporting. Trial- and outcome-specific supporting evidence is provided in Table 5.

### 3.4. Effects on Pain and Disability

Key characteristics and outcomes of the included trials are summarized in Table 4, with quantitative between-group findings presented in Supplementary Table S2. Across the five independent RCT cohorts (reported in six publications), exercise-based interventions generally produced greater improvements in pain-related outcomes (i.e., pain intensity and/or pain-related disability) and neck-related disability compared with minimal intervention or advice alone [8,9,10,17,27,28]. When exercise was compared with other active treatments, between-group effects were variable. For instance, Thompson et al. [27] reported mean changes in disability (NPQ) of −7.2 vs. −10.2 points, with no significant between-group difference, although pain decreased by −1.0 vs. −2.2 NPRS points (*p* < 0.05). Conversely, Javdaneh et al. [17] reported a significant between-group effect on pain of −2.56 points (95% CI −3.32 to −1.80; *p* = 0.019). Overall, these findings indicate heterogeneous effects across active comparators rather than a consistently superior effect of exercise-based interventions.

Overall, between-group effects varied across active comparators, and several comparisons did not reach statistical significance. These non-significant findings should not be interpreted as evidence of equivalence or non-inferiority, as the included trials were not designed or powered for equivalence or non-inferiority testing. Therefore, the relative effectiveness of exercise-based interventions compared with other active treatments remains uncertain.

### 3.5. Effects on Kinesiophobia

Kinesiophobia was assessed using the Tampa Scale for Kinesiophobia (TSK), a validated self-report measure of fear of movement/(re)injury [16]. Specifically, the included studies used either the original 17-item TSK or its shortened 11-item version (TSK-11) [9,10,17,27,28]. The TSK-11 was developed by removing six psychometrically weaker items from the original scale and has demonstrated reliability and validity comparable to the full TSK, while reducing respondent burden [33].

In these studies, exercise-based interventions, particularly when combined with education or cognitive-behavioral strategies, tended to reduce the fear of movement over time within groups. However, between-group differences compared with control or alternative treatments were modest and not consistently statistically significant. Given the small number of trials and heterogeneity in intervention content and follow-up duration, the overall evidence regarding the impact of exercise on kinesiophobia in chronic WAD remains limited and inconclusive.

As such, although kinesiophobia was defined as a secondary outcome in the present review, evidence supporting clear between-group effects remains exploratory, as only a limited number of trials demonstrated statistically and clinically meaningful differences. Nevertheless, fear of movement represents a key construct in chronic WAD, with potential implications for disability persistence and treatment response. The observed trends suggest that kinesiophobia may be a relevant mechanism-based target for future trials, particularly within multimodal and biopsychosocial exercise-based interventions.

### 3.6. Adverse Events

Adverse events were not systematically reported across the included studies. Where safety information was provided, no serious adverse events attributable to the exercise interventions were reported. Given the limited and inconsistent reporting, however, no firm conclusions regarding the safety profile of the interventions can be drawn.

### 3.7. Certainty of Evidence

Using the GRADE framework, certainty of evidence was judged low for pain and neck-related disability and very low for kinesiophobia. Certainty was downgraded primarily because of risk-of-bias concerns, inconsistency across interventions and comparators, imprecision related to the small number of independent RCT cohorts, and the limited availability of kinesiophobia data. These ratings support cautious interpretation of the observed treatment effects.

## 4. Discussion

### 4.1. Summary of the Main Findings

This systematic review synthesized six reports from five independent RCT cohorts, representing up to 635 unique participants, evaluating therapeutic exercise for adults with chronic WAD. Across trials, exercise-based programmes generally produced larger improvements in pain and neck-related disability than minimal or non-active comparators (e.g., advice or education alone). When compared with other active interventions, between-group effects were variable and statistically inconclusive; therefore, the comparative effectiveness of exercise versus active treatments remains uncertain. Evidence on kinesiophobia was limited and mixed, with clearer between-group improvements reported mainly in trials that combined exercise with education or behavioral components. The GRADE assessment rated the certainty of evidence as low for pain and disability and very low for kinesiophobia, emphasizing that the observed benefits should be interpreted cautiously.

### 4.2. Comparison with the Previous Literature

Our findings are broadly consistent with, but also extend, previous systematic reviews. Chrcanovic et al. reported short-term improvements in neck pain and medium-term improvements in disability with exercise therapy for WAD, while emphasizing the weakness and heterogeneity of the evidence [19]. Griffin et al. found no clinically meaningful long-term advantage of general exercise programmes over minimal-intervention comparators [20]. More recently, Muñoz-Bustos et al. found that combining education with exercise produced, at most, small effects on pain that were not clinically important and no clear benefit for disability, with very-low-certainty evidence [21]. These findings are consistent with our observation that exercise-based interventions may be beneficial compared with minimal or non-active care, whereas comparative effectiveness against other active approaches remains uncertain. The present review adds an explicit consideration of kinesiophobia and distinguishes reports from independent randomized cohorts.

### 4.3. Potential Mechanisms

Exercise may contribute to improvements in chronic WAD through neuromuscular adaptations and changes in pain processing [4,8,14]. When combined with education and graded exposure or behavioral strategies, it may also help reduce fear of movement and improve coping strategies [9,10,17].

Central neuroplastic mechanisms may also hypothetically contribute to the clinical effects of therapeutic exercise in chronic WAD. WAD-specific evidence indicates altered nociceptive processing and central hyperexcitability in some individuals with chronic WAD [34,35,36]; however, the trials included in the present review did not directly assess neuroplastic changes or establish a causal relationship between such mechanisms and improvements in pain or disability. It may therefore be hypothesized that repeated, task-specific, and progressively challenging movement could influence sensorimotor processing and pain-related responses through experience-dependent mechanisms. These potential central effects remain biologically plausible but unconfirmed and should be regarded strictly as hypotheses requiring direct investigation using appropriate neurophysiological or neuroimaging outcomes.

The mechanisms discussed in this section were not directly tested by the trials included in this review and should therefore be interpreted as hypotheses that contextualize the observed clinical effects rather than as demonstrated mediators of treatment response. In chronic WAD, exercise combined with education, graded exposure, or behavioral strategies may influence movement confidence, fear-related cognitions, and sensorimotor function, but causal mediation by central neuroplastic mechanisms has not been established [34,35,36].

Pain, disability, and kinesiophobia should not be regarded as independent outcomes in chronic WAD, but rather as interacting components of a multidimensional clinical process. Persistent pain may promote fear of movement and avoidance behaviors, particularly when movement is perceived as potentially harmful or capable of causing reinjury. In turn, kinesiophobia may reduce physical activity and engagement in rehabilitation, contributing to impaired motor function, deconditioning, and persistence of disability. Disability itself may further reinforce pain-related fear by limiting daily activities and reducing confidence in movement, thereby establishing a potentially self-perpetuating pain–fear–avoidance–disability cycle [12,16]. From this perspective, therapeutic exercise may act not only through improvements in strength, mobility, and sensorimotor control, but also through repeated exposure to safe movement, potentially increasing movement confidence and reducing fear-related avoidance. This interaction may help explain why interventions combining exercise with education or behavioral components appear particularly relevant in patients in whom kinesiophobia contributes substantially to disability.

### 4.4. Implications for Practice

Therapeutic exercise can be considered within rehabilitation programmes for chronic WAD, particularly when the comparator is minimal care. Given the heterogeneity of exercise content and delivery across trials, clinicians should avoid assuming a single “best” protocol, tailoring instead exercise to individual presentation, tolerance, and goals within a biopsychosocial framework. In this scenario, some indications may arise from the following:

- Population: adults with chronic WAD (symptoms > 3 months) managed in outpatient rehabilitation settings;
- Intervention content: neck-specific or scapular/neck exercises delivered alone or as part of multimodal programmes; adding education and behavioral components may be reasonable when the fear of movement is prominent;
- Dose and delivery (as tested in included trials): programmes ranged from 4-week protocols to 6-week supervised schedules (three sessions/week) and combined education plus exercise schedules (4 education sessions plus 8 exercise sessions);
- Comparator nuance: effects were more consistent versus minimal care; comparisons versus active treatments were heterogeneous and frequently statistically inconclusive, and should not be interpreted as evidence of equivalence or non-inferiority;
- Safety: adverse events were not systematically reported; where safety information was available, no serious intervention-related adverse events were reported, precluding firm conclusions regarding safety.

### 4.5. Strengths and Limitations

Strengths of this review include the exclusive inclusion of RCTs and the complementary use of the PEDro scale and the Cochrane RoB 2 tool, together with a formal GRADE assessment of certainty of evidence [30,31,32]. PEDro was retained to facilitate comparison with the physiotherapy literature, whereas RoB 2 provided an outcome-focused assessment of potential bias and GRADE allowed the overall confidence in the body of evidence to be explicitly characterized.

Limitations include clinical and methodological heterogeneity, which precluded meta-analysis, and the lack of prospective protocol registration. The absence of a prospectively registered protocol prevents independent verification of the a priori eligibility criteria, outcome hierarchy, and planned synthesis methods and therefore increases uncertainty regarding potential selective eligibility or outcome reporting at the review level. Although the methods and outcome hierarchy are reported transparently in the present manuscript, post hoc modifications cannot be fully excluded. The small number of independent trials limited formal assessment of publication bias and reduced the precision of GRADE judgements. The limited evidence base also constrains generalizability, and the findings should therefore be interpreted cautiously rather than as definitive estimates of treatment effectiveness. In addition, RoB 2 assessments necessarily relied on the completeness of reporting in the published articles, and several domains remained subject to some concerns. The broadened search did not restrict retrieval by kinesiophobia or any other outcome; nevertheless, limitations inherent to database indexing and reporting may have resulted in missed records. In addition, mutually exclusive numerical reason codes for the ten full-text exclusions were not retained in the original screening log. To improve PRISMA transparency at revision, reason-specific counts were retrospectively reconstructed from the available screening notes; these counts should therefore be considered approximate and are reported as such rather than presented as prospectively recorded data.

### 4.6. Future Research

Future RCTs should integrate standardized outcome measures, longer follow-up periods, and systematic assessment of psychological constructs (such as kinesiophobia and pain-related fear) [9,10,12,17,18]. Future systematic reviews in this field should also be prospectively registered (e.g., in PROSPERO) to enhance transparency, methodology, and reproducibility.

## 5. Conclusions

Therapeutic exercise may improve pain and disability in adults with chronic WAD compared with minimal or non-active interventions; however, the certainty of this evidence is low. Its added benefit over other active treatments remains uncertain and evidence regarding effects on kinesiophobia is still limited and inconsistent. Exercise-based rehabilitation should be delivered within a customized biopsychosocial framework, and further high-quality trials are needed to clarify which components and delivery characteristics provide the greatest benefit. From a clinical perspective, clinicians should prioritize individually tailored exercise programmes within a biopsychosocial framework, particularly when fear of movement is prominent, rather than relying on uniform or purely biomechanical exercise prescriptions.

## Figures and Tables

**Table 1:** Search strings used.

Database	Search Strategy	Search Fields	Filters/Limits
PubMed	(“Whiplash Injuries”[MeSH] OR whiplash*[Title/Abstract] OR “whiplash-associated disorder*”[Title/Abstract]) AND (“Exercise Therapy”[MeSH] OR exercise*[Title/Abstract] OR rehabilitation[Title/Abstract])	MeSH Terms and Title/Abstract	None
PEDro	whiplash AND exercise	Title and Abstract	Clinical trials
Cochrane Library	(whiplash OR “whiplash-associated disorder”) AND (exercise OR rehabilitation)	Title, Abstract, Keywords	Trials

## Data Availability

Not applicable.

## Supplementary Materials

The following supporting information can be downloaded at: https://www.mdpi.com/article/10.3390/diseases14100366/s1, Table S1: PRISMA 2020 Checklist (from Page MJ, et al. The PRISMA 2020 statement: an updated guideline for reporting systematic reviews. BMJ 2021;372:n71. Doi: 10.1136/bmj.n71) [22]; Table S2: Quantitative between-group findings for primary pain/disability outcomes [8,9,10,17,27,28].

## Abbreviations

The following abbreviations are used in this manuscript:

BDI-II	Beck Depression Inventory-II
EQ-5D	EuroQol-5 Dimension
GPE	Global Perceived Effect
GRADE	Grading of Recommendations Assessment, Development and Evaluation
HAD	Hospital Anxiety and Depression scale
NDI	Neck Disability Index
NPQ	Northwick Park Neck Pain Questionnaire
NPRS	Numeric Pain Rating Scale
NRS	Numerical rating scale
NSE	Neck-specific exercise
NSEB	Neck-specific exercise with a behavioral approach
PCS	Pain Catastrophizing Scale
PDI	Pain Disability Index
PEDro	Physiotherapy Evidence Database
PICO	Population–Intervention–Comparison–Outcome
PPA	Prescribed physical activity
PRISMA	Preferred Reporting Items for Systematic Reviews and Meta-Analyses
PSFS	Patient-Specific Functional Scale
RCT	Randomized controlled trial
RoB 2	Cochrane Risk of Bias 2
SF-36 PCS	Short Form-36 Physical Component Summary
SWiM	Synthesis Without Meta-analysis
TSK	Tampa Scale for Kinesiophobia
TSK-11	11-item Tampa Scale for Kinesiophobia
VAS	Visual analogue scale
WAD	Whiplash-associated disorders
