# Supplementary material for: Therapeutic Exercise for Chronic Whiplash-Associated Disorders: A Systematic Review of Randomized Controlled Trials on Pain, Disability, and Kinesiophobia
Source: Diseases. 2026 Oct 2;14(10):366. doi: 10.3390/diseases14100366 (PMC13703367; doi:10.3390/diseases14100366)
Supplement: Supplementary file 1 [file diseases-14-00366-s001.zip › diseases-4535085-supplementary.pdf]

Table S1. PRISMA 2020 Checklist (from Page MJ, et al. The PRISMA 2020 statement: an updated guideline for reporting systematic reviews. BMJ 2021;372:n71. Doi: 10.1136/bmj.n71). [22]

Section and Topic	Item #	Checklist item	Location where item is reported
**TITLE**			
Title	1	Identify the report as a systematic review.	Title
**ABSTRACT**			
Abstract	2	See the PRISMA 2020 for Abstracts checklist.	Abstract
**INTRODUCTION**			
Rationale	3	Describe the rationale for the review in the context of existing knowledge.	Introduction
Objectives	4	Provide an explicit statement of the objective(s) or question(s) the review addresses.	Introduction (PICO objective)
**METHODS**			
Eligibility criteria	5	Specify the inclusion and exclusion criteria for the review and how studies were grouped for the syntheses.	Methods, Section 2.2; Section 2.7
Information sources	6	Specify all databases, registers, websites, organisations, reference lists and other sources searched or consulted to identify studies. Specify the date when each source was last searched or consulted.	Methods, Section 2.3
Search strategy	7	Present the full search strategies for all databases, registers and websites, including any filters and limits used.	Methods, Section 2.3; Table 1
Selection process	8	Specify the methods used to decide whether a study met the inclusion criteria of the review, including how many reviewers screened each record and each report retrieved, whether they worked independently, and if applicable, details of automation tools used in the process.	Methods, Section 2.4
Data collection process	9	Specify the methods used to collect data from reports, including how many reviewers collected data from each report, whether they worked independently, any processes for obtaining or confirming data from study investigators, and if applicable, details of automation tools used in the process.	Methods, Section 2.4
Data items	10a	List and define all outcomes for which data were sought. Specify whether all results that were compatible with each outcome domain in each study were sought (e.g. for all measures, time points, analyses), and if not, the methods used to decide which results to collect.	Methods, Section 2.6
	10b	List and define all other variables for which data were sought (e.g. participant and	Methods, Section 2.4; Table 4

Section and Topic	Item #	Checklist item	Location where item is reported
		intervention characteristics, funding sources). Describe any assumptions made about any missing or unclear information.	
Study risk of bias assessment	11	Specify the methods used to assess risk of bias in the included studies, including details of the tool(s) used, how many reviewers assessed each study and whether they worked independently, and if applicable, details of automation tools used in the process.	Methods, Section 2.5; Results, Section 3.3; Table 5
Effect measures	12	Specify for each outcome the effect measure(s) (e.g. risk ratio, mean difference) used in the synthesis or presentation of results.	Methods, Section 2.7; Results, Section 3.4; Supplementary Table S2
Synthesis methods	13a	Describe the processes used to decide which studies were eligible for each synthesis (e.g. tabulating the study intervention characteristics and comparing against the planned groups for each synthesis (item #5)).	Methods, Sections 2.2 and 2.7
	13b	Describe any methods required to prepare the data for presentation or synthesis, such as handling of missing summary statistics, or data conversions.	Methods, Section 2.7
	13c	Describe any methods used to tabulate or visually display results of individual studies and syntheses.	Methods, Section 2.7; Tables 4–5; Supplementary Table S2
	13d	Describe any methods used to synthesize results and provide a rationale for the choice(s). If meta-analysis was performed, describe the model(s), method(s) to identify the presence and extent of statistical heterogeneity, and software package(s) used.	Methods, Section 2.7
	13e	Describe any methods used to explore possible causes of heterogeneity among study results (e.g. subgroup analysis, meta-regression).	Not applicable; no formal subgroup analysis or meta-regression
	13f	Describe any sensitivity analyses conducted to assess robustness of the synthesized results.	Not applicable; no sensitivity analysis performed
Reporting bias assessment	14	Describe any methods used to assess risk of bias due to missing results in a synthesis (arising from reporting biases).	Methods, Section 2.7; Discussion, Section 4.5
Certainty assessment	15	Describe any methods used to assess certainty (or confidence) in the body of evidence for an outcome.	Methods, Section 2.8; Results, Section 3.7; Discussion, Sections 4.1 and 4.5
**RESULTS**			
Study selection	16a	Describe the results of the search and selection process, from the number of records identified in the search to the number of studies included in the review, ideally using a flow diagram.	Results, Section 3.1; Figure 1
	16b	Cite studies that might appear to meet the inclusion criteria, but which were excluded, and explain why they were excluded.	Results, Section 3.1; Figure 1 (reason-specific counts

Section and Topic	Item #	Checklist item	Location where item is reported
			retrospectively reconstructed and explicitly identified as approximate); Discussion, Section 4.5
Study characteristics	17	Cite each included study and present its characteristics.	Results, Section 3.2; Table 4
Risk of bias in studies	18	Present assessments of risk of bias for each included study.	Results, Section 3.3; Table 5
Results of individual studies	19	For all outcomes, present, for each study: (a) summary statistics for each group (where appropriate) and (b) an effect estimate, and its precision (e.g. confidence/credible interval), ideally using structured tables or plots.	Results, Sections 3.4–3.5; Table 4; Supplementary Table S2
Results of syntheses	20a	For each synthesis, briefly summarise the characteristics and risk of bias among contributing studies.	Results, Sections 3.2–3.4; Tables 4–5
	20b	Present results of all statistical syntheses conducted. If meta-analysis was done, present for each the summary estimate and its precision (e.g. confidence/credible interval) and measures of statistical heterogeneity. If comparing groups, describe the direction of the effect.	Not applicable; no meta-analysis performed. SWiM synthesis reported in Results, Section 3.4 and Supplementary Table S2
	20c	Present results of all investigations of possible causes of heterogeneity among study results.	Not applicable; heterogeneity addressed descriptively in SWiM synthesis
	20d	Present results of all sensitivity analyses conducted to assess the robustness of the synthesized results.	Not applicable; no sensitivity analysis performed
Reporting biases	21	Present assessments of risk of bias due to missing results (arising from reporting biases) for each synthesis assessed.	Discussion, Section 4.5; formal assessment not feasible because of the small evidence base
Certainty of evidence	22	Present assessments of certainty (or confidence) in the body of evidence for each outcome assessed.	Results, Section 3.7; Discussion, Sections 4.1 and 4.5 (GRADE certainty assessment)
**DISCUSSION**			
Discussion	23a	Provide a general interpretation of the results in the context of other evidence.	Discussion, Sections 4.1–4.4
	23b	Discuss any limitations of the evidence included in the review.	Discussion, Section 4.5
	23c	Discuss any limitations of the review processes used.	Discussion, Section 4.5
	23d	Discuss implications of the results for practice, policy, and future research.	Discussion, Sections 4.4 and 4.6

Section and Topic	Item #	Checklist item	Location where item is reported
**OTHER INFORMATION**			
Registration and protocol	24a	Provide registration information for the review, including register name and registration number, or state that the review was not registered.	Methods, Section 2.1: review not prospectively registered
	24b	Indicate where the review protocol can be accessed, or state that a protocol was not prepared.	Methods, Section 2.1: no prospectively registered protocol
	24c	Describe and explain any amendments to information provided at registration or in the protocol.	Methods, Sections 2.1 and 2.3; Discussion, Section 4.5 (absence of prospective protocol and review-stage search amendment transparently described)
Support	25	Describe sources of financial or non-financial support for the review, and the role of the funders or sponsors in the review.	Funding statement
Competing interests	26	Declare any competing interests of review authors.	Conflicts of Interest statement
Availability of data, code and other materials	27	Report which of the following are publicly available and where they can be found: template data collection forms; data extracted from included studies; data used for all analyses; analytic code; any other materials used in the review.	Data Availability Statement

This work is licensed under CC BY 4.0. To view a copy of this license, visit https://creativecommons.org/licenses/by/4.0/

Note: For PRISMA item 16b, individual excluded full-text reports could not be listed with prospectively recorded mutually exclusive reason codes because the original screening log did not preserve that level of detail. Reason-specific counts were reconstructed retrospectively from the available screening notes, explicitly labelled as approximate in the manuscript, and acknowledged as a review-process limitation.

Table S2. Quantitative between-group findings for primary pain/disability outcomes.

Report [ref]	Outcome	Time point	Between-group effect estimate	95% CI	*p*-value
Javdaneh et al., 2020 [17]	Pain (VAS)	6 weeks	−2.56 points	−3.32 to −1.80	0.019
Thompson et al., 2016 [27]	Disability (NPQ)	6 months	NR	NR	NS
Thompson et al., 2016 [27]	Pain (NPRS)	6 months	NR	NR	<0.05
Peterson et al., 2015 [10]	Pain after neck-muscle endurance testing	3–6 months	NR	NR	0.03 (overall group difference)
Overmeer et al., 2016 [9]	Pain-related disability (PDI)	3–24 months	Between-group estimate NR; longitudinal model reported	NR	See original report
Ris et al., 2016 [28]	Disability (NDI)	4 months	NR	NR	NR
Sarig Bahat et al., 2018 [8]	Pain/disability	1 and 3 months	NR for WAD-specific subgroup	NR	NR

NR = not reported or not validly derivable from the published report for the eligible comparison; NS = not statistically significant. No unsupported effect estimates were reconstructed. Sarig Bahat et al. enrolled a mixed chronic-neck-pain population; WAD-specific quantitative effects were not separately reported and should therefore be interpreted cautiously. These data support the SWiM synthesis reported in Section 3.4 of the main manuscript.

References

8. Sarig Bahat, H.; Weiss, P.L.; Sprecher, E.; Treleaven, J. Remote kinematic training for patients with chronic neck pain: A randomized controlled trial. *Eur. Spine J.* **2018**, *27*, 1309–1323.

9. Overmeer, T.; Peterson, G.; Landén Ludvigsson, M.; Peolsson, A. The effect of neck-specific exercise with or without a behavioral approach on psychological factors in chronic whiplash-associated disorders: A randomized controlled trial with a 2-year follow-up. Medicine **2016**, *95*, e4430.

10. Peterson, G.E.; Landén Ludvigsson, M.H.; O'Leary, S.P.; Dedering, Å.M.; Wallman, T.; Jönsson, M.I.N.; Peolsson, A.L.C. The effect of three different exercise approaches on neck muscle endurance, kinesiophobia, exercise compliance and patient satisfaction in chronic whiplash. *J. Manip. Physiol. Ther.* **2015**, *38*, 465–476. https://doi.org/10.1016/j.jmpt.2015.06.011.

17. Javdaneh, N.; Letafatkar, A.; Shojaedin, S.; Hadadnezhad, M. Scapular exercise combined with cognitive functional therapy is more effective than scapular exercise alone in reducing chronic neck pain and kinesiophobia: A randomized controlled trial. *Clin. Rehabil.* **2020**, *34*, 1385–1395.

22. Page, M.J.; McKenzie, J.E.; Bossuyt, P.M.; Boutron, I.; Hoffmann, T.C.; Mulrow, C.D.; Shamseer, L.; Tetzlaff, J.M.; Akl, E.A.; Brennan, S.E.; et al. The PRISMA 2020 statement: An updated guideline for reporting systematic reviews. *BMJ* **2021**, *372*, n71.

27. Thompson, D.P.; Oldham, J.A.; Woby, S.R. Does adding cognitive-behavioural physiotherapy to exercise improve outcome in patients with chronic neck pain? A randomised controlled trial. *Physiotherapy* **2016**, *102*, 170–177. https://doi.org/10.1016/j.physio.2015.04.008.

28. Ris, I.; Søgaard, K.; Gram, B.; Agerbo, K.; Boyle, E.; Juul-Kristensen, B. Does a combination of physical training, specific exercises and pain education improve health-related quality of life in patients with chronic neck pain? A randomised control trial with a 4-month follow up. *Man. Ther.* **2016**, *26*, 132–140. https://doi.org/10.1016/j.math.2016.08.004.
